# Physical Growth of Patients with Hereditary Tyrosinaemia Type I: A Single-Centre Retrospective Study

**DOI:** 10.3390/nu13093070

**Published:** 2021-08-31

**Authors:** Ozlem Yilmaz, Anne Daly, Alex Pinto, Catherine Ashmore, Sharon Evans, Girish Gupte, Richard Jackson, Nurcan Yabanci Ayhan, Anita MacDonald

**Affiliations:** 1 Birmingham Women’s and Children’s Hospital, Birmingham B4 6NH, UK; o.yilmaz@ybu.edu.tr (O.Y.); a.daly3@nhs.net (A.D.); alex.pinto@nhs.net (A.P.); catherine.ashmore@nhs.net (C.A.); evanss21@me.com (S.E.); girishgupte@nhs.net (G.G.); 2 Department of Nutrition and Dietetics, Faculty of Health Sciences, Ankara Yildirim Beyazit University, Ankara 06760, Turkey; 3 Department of Nutrition and Dietetics, Faculty of Health Sciences, Ankara University, Ankara 06290, Turkey; nyabanci@gmail.com; 4 Cancer Research UK Liverpool Cancer Trials Unit, University of Liverpool, Liverpool L69 3GL, UK; R.J.Jackson@liverpool.ac.uk

**Keywords:** tyrosinaemia, growth, physical outcomes, weight, height, BMI, phenylalanine, tyrosine, diet

## Abstract

In a retrospective review, we aimed to assess long-term growth in 17 patients (*n* = 11 males) with hereditary tyrosinaemia type I (HTI). Median age at assessment was 15.6 years (5.7–26.6 years) and median age at diagnosis was 1 month (range: 0–16 months), with 35% (*n* = 6/17) symptomatic on presentation. From the age of 8 years, there was a noticeable change in median height, weight, and body-mass-index [BMI]-z-scores. Median height-for-age z-scores were consistently ≤ −1 (IQR −1.6, −0.5) during the first 8 years of life but increased with age. Weight-for-age z-scores ranged between −1 to 0 (IQR −1.2, 0.1) in the first 8 years; then increased to > 0.5 (IQR −0.3, 1.3) by age 16 years, and BMI-for-age z-scores ranged from 0 to 1 (IQR −0.7, 1.3) up to 8 years, and >1 (IQR −0.2, 1.9) until 16 years. The percentage of overweight and obesity was lowest in children aged < 5 years, and consistently > 40% in patients aged between 7 to 16 years. The prescribed total protein intake was associated with improved height growth (p < 0.01). Impaired growth in early life improved with age achieving normal population standards. Further studies are needed to investigate factors that influence growth outcome in HTI patients.

## 1. Introduction

Following the successful treatment of hereditary tyrosinaemia type I (HTI) with nitisinone (2-(2-nitro-4-trifluoromethyl-benzoyl)-1,3-cyclohexanedione, NTBC) [1,2,3], and a low phenylalanine/tyrosine diet, the management of HTI has radically altered, with a significant decrease in the number of patients needing lifesaving liver transplantations [1,2,4]. Impaired neurocognition is still an emerging clinical problem, although its underlying pathophysiology remains unclear [2,5,6,7,8,9,10,11,12].

The dietary treatment for HTI shares similar principles with other amino acid disorders. It consists of a low protein diet supplemented with a low or free tyrosine/phenylalanine protein substitute usually supplemented with vitamins, minerals, and trace elements to meet nutritional requirements. The main aims of dietary treatment are to maintain tyrosine and phenylalanine concentrations within a defined target therapeutic range and promote normal growth and development. Although there are no agreed guidelines on blood tyrosine and phenylalanine concentrations for HTI, tyrosine is commonly maintained between 200-400 µmol/L and phenylalanine > 30 µmol/L and ideally ≥ 50 [1,13,14,15]. In HTI, low blood phenylalanine concentrations are a common finding, particularly in the early years of life and phenylalanine supplementation is necessary [13,15,16,17]. The exact cause of low blood phenylalanine concentrations is unknown. However, in a phenylketonuria (PKU) animal study, pharmacological inhibition of tyrosine metabolism with NTBC increased blood and brain tyrosine, and lowered phenylalanine concentrations, suggesting that NTBC may decrease phenylalanine concentrations [18].

Despite considerable experience with dietary treatment in HTI, precise nutritional requirements are not well-defined. It has been reported that patients with HTI tolerate more natural protein with increasing age without compromising metabolic control [19,20]. In PKU, there is some evidence to suggest that maximizing natural protein intake may improve height growth and body composition parameters [21,22]. In HTI, longitudinal growth was not reported prior to NTBC introduction due to high mortality rates or early liver transplantation [17], but since NTBC introduction, data on growth is scarce and inconclusive [23]. Growth is an important outcome and measures in part, the efficacy of dietary treatment. Therefore, the aim of this audit was to assess long-term growth of HTI patients treated with lifelong protein restriction and NTBC from the time of diagnosis. 

## 2. Materials and Methods

### 2.1. Project Design 

In this longitudinal retrospective review, data was collected on patients with HTI followed at Birmingham Children’s Hospital, UK from the time of diagnosis until transition to the adult services (between the age of 16 and 18 years). Data on clinical presentation, diagnosis, anthropometry and prescribed dietary intake were collected. Eligibility criteria included: (1) patients prescribed a low protein diet supplemented with phenylalanine/tyrosine free protein substitute and (2) NTBC treatment since diagnosis. Patients aged under 5 years (at the time of data collection), post-liver transplant or patients with co-morbidities that may affect dietary management (e.g., diabetes, liver cancer) were excluded.

### 2.2. NTBC and succinylacetone concentrations

Serum NTBC and plasma succinylacetone concentrations taken in clinic were recorded for each subject by age.

### 2.3. Dietary prescriptions

Annual prescribed total protein, natural protein and protein equivalent from protein substitutes were obtained for each patient. Data on phenylalanine supplementation was also collected. The dietary management in our centre has been previously described [19].

### 2.4. Anthropometry 

Annual weight (kg), height (cm) and body mass index (BMI) (kg/m^2^) were collected from medical and dietetic records. The measurements selected were the closest to their birthday (±6 months). BMI was calculated by the ratio of weight in kilograms to height in meters squared. 

Weight, height, and BMI for-age z-scores were also calculated. Definitions to characterize BMI-for-age z-score according to WHO classification [24,25,26] are given in Table 1.

### 2.5. Ethical statement

This project was registered as an audit (CARMS 30957) and conducted in accordance with ethical principles with respect for the confidentiality of participants and in line with the Data Protection Act 2000. Age-appropriate informed consent was given by caregivers/patients and assent by children/adolescents to participate in this study.

### 2.6. Statistics

Analyses were performed using SPSS^®^ (version 22, SPSS Inc., Chicago, IL, USA) and R (version 3, R Foundation for Statistical Computing, Vienna, Austria). Continuous data were presented as medians (first and third quartile) and categorical data were presented as percentages. Linear statistical modelling for weight, height and BMI are compared with age as a categorized covariate (<8y vs. >8y). Correlations between anthropometric parameters (weight, height, and BMI) and prescribed protein intake were performed using multivariable linear regression. A p-value < 0.05 was considered statistically significant throughout.

## 3. Results

### 3.1. Patients

Seventeen patients with HTI (11 males) were assessed. The median age was 15.6 years (range: 5.7–26.6 years) at the final assessment. And at diagnosis was 1 month (range: 0 to 16 months). Patients’ ethnic origin was Pakistani Asian (*n* = 8/17, 47%), European/Caucasian (*n* = 7/17, 41%), Arabic (*n* = 1/17, 6%) and Indian (*n* = 1/17, 6%). Fifty-nine percent (*n* = 10/17) of patients were from consanguineous marriages. Seven patients (41%) were diagnosed by sibling/family screening and 6 patients (35%) presented clinically with acute liver failure. Three patients (18%) were detected due to raised tyrosine concentrations on routine newborn screening for PKU and one patient (6%) was from a country with universal HTI newborn screening.

### 3.2. Dietary prescription

The total protein, natural protein, and protein equivalent intakes from protein substitutes prescribed by age are given in Table 2. The median prescribed total protein intake (including natural protein and protein equivalent from protein substitutes) remained consistently above 2 g/kg/day (IQR 2.2 to 3.0 g/kg/day) in the first 8 years of life decreasing to 1.9 g/kg/day (IQR 1.7 to 2.1 g/kg/day) at 9 years and to 1.3 g/kg/day (IQR 1.2 to 1.4 g/kg/day) at 16 years. 

The median prescribed natural protein intake remained consistent at a median of 0.4 g/kg/day (IQR 0.3 to 0.5 g/kg/day) from the first year until the age of 16 years. When expressed as g/day, the median prescribed natural protein intake increased from 4 g/day (IQR 3 to 6 g/day) at first year to 30 g/day (IQR 27 to 32 g/day) at 16 years.

The median prescribed protein equivalent intake from protein substitutes decreased with age: 2.8 g/kg/day (IQR 2.3 to 3.1 g/kg/day) in the first year; 1.0 g/kg/day (IQR 0.9 to 1.3 g/kg/day) from 12 to 15 years, and 0.7 g/kg/day (IQR 0.6 to 0.9 g/kg/day) at 16 years.

### 3.3. Anthropometric Parameters

**Figure 1:**
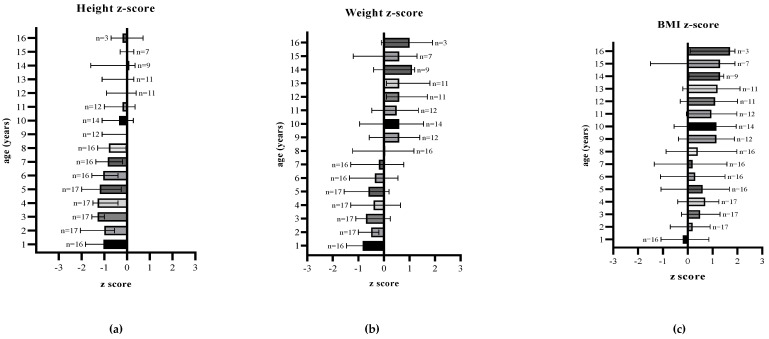
(**a**) Median height-for-age, (**b**) median weight-for-age, and (**c**) median BMI-for-age z-scores of patients with HTI from first year to 16 years of age.

Table 2 and Figure 1 shows anthropometric characteristics of patients by age.

Height for age z-score: median z-score was ≤ −1 (IQR −1.6, −0.4) for the first 8 years of age, but showed an improvement over time and remained predominantly at 0 (IQR −1.0, 0.3) between 9 and 16 years of age. 

Weight for age z-score: median z-score ranged between −1 and 0 (−1.2, 0.1) for the first 8 years of age and then remained consistently ≥0.5 (IQR −0.3, 1.3) until 16 years of age (end of follow up), although the number of patients followed up in each age group decreased with age.

BMI for age z-score: from the age of 1 to 8 years of age, median z-scores remained between 0 and 1 (IQR −0.7, 1.3), and then predominantly >1 (IQR −0.2, 1.9) in older children.

Statistical modelling for weight, height and BMI compared prescribed total, natural and protein equivalent intake with age. An increase in prescribed total protein was significantly associated with an increase in height (r = 0.5, *p* < 0.01). No other correlations were found between anthropometry, natural protein, or protein equivalent intake. For weight, height, and BMI a linear relationship with age was observed.

### 3.4. Overweight and obesity by age

Patients aged < 5 years were less overweight (range 0% to 12%) or obese (range 0% to 6%) compared to those > 5 years. The percentage of patients who were overweight and obese increased from 41% (*n* = 7/17) at 5 years to 67% (*n* = 2/3) at 16 years. The median percentage of overweight and obesity between 9 and 16 years of age was 43% (range 25–67%) and 21% (range 0–27%), respectively. However, there were fewer patient numbers studied in the older age groups (Table 3).

### 3.5. Serum NTBC and plasma succinylacetone concentrations

From 1 to 16 years of age, the median blood NTBC concentration of patients was 45 μmol/L (range 28 to 52 μmol/L); the normal recommended therapeutic reference range is 40–60 μmol/L [27]. The median plasma succinylacetone concentrations were below the detectable limits (<0.05 units), as recommended (or at least within the limits of normal established by the reference laboratory) [14]. Monitoring of serum NTBC and succinylacetone concentrations are used to assess drug adherence and dosage.

## 4. Discussion

The two key observations from this longitudinal retrospective review on growth in HT1 in children treated with NTBC and a low tyrosine diet were: (1) impaired growth in early childhood associated with a negative height and weight z-score, and (2) a statistically significant correlation between height and prescribed total protein intake. From 8 years of age there was a noticeable change in height, and median height-for-age z-scores were close or equal to the population norm by 16 years of age. Weight and BMI z-scores also accelerated from 8 years of age, and overweight and obesity rates were higher than the UK healthy population [28].

Growth is a complex physiological process regulated by genetic, hormonal, and environmental factors [29,30]. Nutrition is the most influential factor affecting growth in infancy, but after this period, nutritional influences become less important, and the effect of growth hormone increasingly affects linear growth [30]. Thirty five percent (n=6) of our cohort presented with acute liver failure, needing short term nutritional support and the impact of this on growth outcome is unknown. A nationwide study from Finland [31] reviewing long term outcomes in HTI reported low median adult height z-scores in both post transplanted (−1.5) and NTBC treated (−1.4) patients. The authors considered several possible factors leading to short stature included ongoing liver disease, low serum NTBC concentrations and complications post transplantation. Low serum NTBC (≤0.54 μmol/L) was also associated with liver transplantation and learning difficulties. In our study, serum plasma NTBC concentrations were close to the lower recommended reference range [27] but unlike Äärelä et. al. [31] sub-optimal linear growth was only observed in the first 8 years regardless of clinical presentation. This suggested that low NTBC was not correlated with growth failure in our study group. 

Phenylalanine deficiency may have also contributed to suboptimal linear growth. In HTI patients, low phenylalanine concentrations associated with a severe natural protein restriction are described [4,13,16,32]. We have previously reported [19] a median of 32% of all blood phenylalanine concentrations below the reference value (≤ 30 μmol/L) in the first year of life. A recent multicentre study [20] reported that dietary relaxation in patients with HTI led to increased phenylalanine concentrations. Phenylalanine is an essential amino acid and low concentrations may be a rate-limiting factor for protein synthesis adversely affecting growth particularly in the early years when lower phenylalanine concentrations have been documented [13]. 

Nutritional requirements of patients with HTI are currently not well defined, but higher protein intakes than WHO/FAO/UNU 2007 safe levels of protein intake are recommended [14,33], accounting for the known inefficient absorption of amino acids from protein substitutes [34]. In PKU, some studies have suggested that higher protein intakes are related to improved linear growth outcomes, although it is unclear which protein source (natural protein, protein equivalent from protein substitutes, or both) has the most influence on physical development [35]. In our cohort, median intakes (g/day) of prescribed total protein were correlated positively with height (*p* < 0.01). 

The results of our study indicated that overweight and obesity rates were higher than the UK general population [28] which show that 28% of healthy children aged 2 to 15 years were overweight or obese. We studied <10 patients in the older age group, and so our results may not be a representative finding. However, our results are similar to a Spanish retrospective study [36], which showed nearly half of the patients (*n* = 24/52, 46%) with HTI were overweight or obese. Preventing this excessive weight and BMI increase is of clinical importance.

The cause of this high incidence of overweight and obesity is unknown and may be due to a variety of factors including ethnicity, unhealthy eating patterns, physical inactivity, a high carbohydrate diet or possibly insulin resistance. In our cohort, 3 children presented with hyperinsulinemic hypoglycemia, one was treated with diazoxide and 2 with additional glucose polymers in early infancy. Hypoglycemia in young infants with HTI is not uncommon [37,38,39,40,41]. The exact cause is undetermined, some describing histological abnormalities of the pancreas with an increased number of islets of Langerhans and hypertrophy [40,42], while others suggest impaired gluconeogenesis due to poor hepatic function and/or reduced hepatic clearance or insufficient glycogen stores [43,44]. Others have suggested toxic metabolites altering intracellular ATP homeostasis or islet cell hyperplasia altering glucose status [38,45]. Not all children had hyperinsulinism, but it is not an uncommon complication in HTI. A comprehensive approach is needed to monitor nutritional and clinical status in HTI patients including assessment of body composition, insulin resistance, triglycerides and excluding non-alcoholic fatty liver disease.

This study has several limitations. Data was collected retrospectively from one single UK centre with a limited subject sample size. Anthropometric measurements were performed by different professionals in routine clinics, although measuring equipment was calibrated according to hospital standards. Dietary management guidelines, protein substitute nutritional composition, and special low protein foods have changed throughout the years, so may have affected dietary patterns and nutritional intake. The prescribed amount of dietary protein may not accurately reflect the actual intake, as many patients did not weigh protein exchanges suggesting an underestimation of natural protein intake. Moreover, there is no data on adherence to protein substitute. We did not collect data on energy intake or ratio of carbohydrate and fat used in the diets which may have explained accelerated weight gain. However, metabolic control was satisfactory and was regularly monitored. We did not compare our cohort with a control group to consider impact of economic status or ethnicity factors on growth outcomes. Parental height was not available, and physical activity data for each subject is unknown.

## 5. Conclusions

We have shown that impaired linear growth in patients with HTI in early childhood, improved over time reaching population norms. However, more research is required to understand which specific factors influenced this change. The actual natural protein intake compared to the theoretical intake is a missing valuable piece of data, particularly as children self-relax their dietary intake as they became older. Similarly, the underlying cause of neurocognitive dysfunction and the cause of overweight and obesity are further areas that need investigation.

## Figures and Tables

**Table 1:** Definitions to characterize BMI-for-age z-score in patients with HTI.

Definitions	BMI-for-Age z-Score	
	<5 Years	5–19 Years
**Overweight**	≥ +2 SD	≥ +1 SD
**Obesity**	≥ +3 SD	≥ +2 SD

Abbreviations: BMI: Body mass index.; SD: Standard Deviation.

**Table 2:** Anthropometric characteristics and prescribed total protein, natural protein and protein equivalent intake from protein substitutes by age.

Age (years)	Number of patients	Height (cm)	Height-for-age z-score	Weight (kg)	Weight-for-age z-score	BMI (kg/m^2^)	BMI-for-age z-score	Protein (g/kg/day)		
								Total	NP	PE from PS
		Median (Q_1,_ Q_3_)								
**1**	16	73.4 (71.6, 75.8)	−1.0 (−1.7, −0.3)	8.9 (8.8, 10.2)	−0.8 (−1.4, −0.2)	16.8 (16.2, 18.6)	−0.2 (−1.0, 0.8)	3.4 (2.9, 3.6)	0.5 (0.3, 0.7)	2.8 (2.3, 3.1)
**2**	17	84.0 (80.7, 87.0)	−1.0 (−2.0, −0.6)	11.7 (11.2, 12.3)	−0.5 (−0.9, −0.2)	16.6 (15.8, 17.5)	0.2 (−0.6, 0.8)	2.8 (2.7, 3.1)	0.5 (0.3, 0.7)	2.4 (2.3, 2.6)
**3**	17	91.7 (90.3, 92.5)	−1.3 (−1.5, −1.0)	13.7 (12.7, 15.0)	−0.7 (−1.1, 0.1)	16.7 (15.8, 17.7)	0.5 (−0.2, 1.2)	2.7 (2.5, 2.9)	0.4 (0.3, 0.5)	2.2 (1.9, 2.6)
**4**	17	99.3 (96.1, 100.3)	−1.3 (−1.5, −0.5)	16.1 (14.4, 17.4)	−0.4 (−1.2, 0.4)	16.6 (16.0, 17.5)	0.7 (0.2, 1.2)	2.5 (2.3, 3.0)	0.4 (0.3, 0.5)	2.1 (1.9, 2.7)
**5**	17	104.9 (102.3, 109.3)	−1.2 (−2.0, −0.3)	17.1 (15.8, 19.4)	−0.6 (−1.5, 0.1)	16.3 (14.4, 17.5)	0.6 (−1.0, 1.5)	2.5 (2.4, 2.9)	0.5 (0.3, 0.6)	2.2 (1.9, 2.4)
**6**	16	111.9 (109.5, 114.9)	−1.1 (−1.5, −0.4)	20.1 (18.5, 22.3)	−0.3 (−1.1, 0.3)	15.9 (15.0, 18.0)	0.3 (−0.5, 1.5)	2.3 (2.0, 2.7)	0.4 (0.4, 0.5)	2.0 (1.6, 2.2)
**7**	16	118.8 (115.8, 121.5)	−0.9 (−1.3, −0.2)	22.9 (19.9, 26.3)	−0.2 (−1.1, 0.7)	15.8 (14.0, 18.4)	0.2 (−1.3, 1.5)	2.3 (2.0, 2.6)	0.4 (0.3, 0.5)	1.9 (1.6, 2.2)
**8**	16	123.5 (121.4, 127.5)	−0.8 (−1.3, −0.2)	25.8 (23.2, 31.4)	0.0 (−0.9, 1.1)	16.5 (14.9, 20.0)	0.4 (−0.6, 1.9)	2.2 (1.9, 2.2)	0.4 (0.3, 0.5)	1.8 (1.5, (1.9)
**9**	12	133.0 (127.0, 134.0)	0.0 (−1.1, 0.0)	32.0 (27.1, 37.1)	0.6 (−0.5, 1.4)	17.2 (15.1, 20.1)	1.1 (−0.1, 1.9)	1.9 (1.7, 2.1)	0.4 (0.3, 0.5)	1.6 (1.4, 1.7)
**10**	14	137.0 (133.8, 140.0)	−0.3 (−1.1, 0.2)	36.6 (29.4, 42.0)	0.6 (−0.6, 1.4)	19.3 (15.7, 22.0)	1.2 (−0.5, 1.9)	1.8 (1.6, 2.2)	0.5 (0.4, 0.6)	1.5 (1.2,1.7)
**11**	12	143.3 (138.9, 146.4)	−0.2 (−1.0, 0.2)	39.3 (34.1, 46.7)	0.5 (−0.2, 1.1)	19.3 (17.8, 21.6)	0.9 (0.3, 1.7)	1.6 (1.5, 2.0)	0.4 (0.3, 0.5)	1.3 (1.1, 1.5)
**12**	11	150.4 (147.1, 154.1)	0.0 (−0.7, 0.4)	47.1 (42.3, 51.0)	0.6 (0.2, 1.3)	20.5 (18.7, 22.8)	1.1 (0.1, 1.8)	1.5 (1.3, 1.9)	0.4 (0.3, 0.5)	1.0 (1.0, 1.4)
**13**	11	154.6 (151.1, 159.7)	0.0 (−0.9, 0.3)	52.3 (49.1, 60.1)	0.6 (0.3, 1.4)	22.1 (19.3, 24.6)	1.2 (0.4, 2.0)	1.4 (1.2, 1.6)	0.3 (0.3, 0.4)	1.0 (0.9, 1.2)
**14**	9	161.8 (151.6, 165.3)	0.1 (−1.6, 0.3)	62.2 (49.3, 63.1)	1.1 (−0.3, 1.2)	22.4 (21.6, 23.1)	1.3 (0.7, 1.4)	1.3 (1.2, 1.6)	0.4 (0.3, 0.4)	1.0 (0.9, 1.2)
**15**	7	166.2 (162.9, 171.5)	0.0 (−0.3, 0.3)	61.9 (53.8, 66.7)	0.6 (−0.5, 1.2)	23.8 (18.7, 24.8)	1.3 (−0.5, 1.7)	1.5 (1.3, 1.7)	0.4 (0.3, 0.5)	1.0 (0.9, 1.2)
**16**	3	172.1 (170.2, 175.6)	−0.2 (−0.4, 0.3)	71.2 (65.6, 78.0)	1.0 (0.5, 1.5)	25.2 (22.7, 25.8)	1.7 (0.9, 1.8)	1.3 (1.2, 1.4)	0.5 (0.3, 0.5)	0.7 (0.6, 0.9)

Abbreviations: Q1: first quartile; Q3: third quartile; BMI: body-mass-index; NP: natural protein, PE: Protein Equivalent; PS: Protein Substitute.

**Table 3:** Percentage of overweight and obesity from first year to 16 years of age.

Age (Years)	Number of Patients	Overweight *n* (%)	Obesity *n* (%)
**1**	16	0 (0)	0 (0)
**2**	17	1 (6)	1 (6)
**3**	17	0 (0)	1 (6)
**4**	17	2 (12)	1 (6)
**5**	17	5 (29)	2 (12)
**6**	16	3 (19)	2 (13)
**7**	16	6 (38)	1 (6)
**8**	16	3 (19)	4 (25)
**9**	12	4 (33)	2 (17)
**10**	14	5 (36)	3 (21)
**11**	12	3 (25)	3 (25)
**12**	11	3 (27)	3 (27)
**13**	11	5 (46)	3 (27)
**14**	9	5 (56)	1 (11)
**15**	7	3 (43)	1 (14)
**16**	3	2 (67)	0 (0)

## Data Availability

The data presented in this study are available on request from the corresponding author.
